# Decentralized Biobanking Pathway to Precision Medicine: Futures Study

**DOI:** 10.2196/73965

**Published:** 2025-12-01

**Authors:** Marielle Gross, Ananya Dewan, Kennedy Sabharwal, Amanda Linkous, M Eifler, William Sanchez, Robert Miller, Mario Macis, Joshua C Rubin

**Affiliations:** 1 Johns Hopkins Berman Institute of Bioethics, Baltimore, MD, United States; 2 de-bi, co, 409 E Baltimore St, Greencastle, PA, 17225, United States, 1 8135416103; 3 Center for Health Justice & Bioethics, Lewis Katz School of Medicine, Temple University, Philadelphia, PA, United States; 4 Johns Hopkins University, Baltimore, MD, United States; 5 University of Kentucky, Lexington, KY, United States; 6 Vanderbilt University, Nashville, TN, United States; 7 Mayo Clinic Comprehensive Cancer Center (Minnesota), Rochester, MN, United States; 8 Indiana University Hospital, Indianapolis, IN, United States; 9 University of Michigan–Ann Arbor, Ann Arbor, MI, United States; 10 Learning Health Community, Arlington, VA, United States; 11 Joseph H. Kanter Family Foundation, Santa Ana, CA, United States

**Keywords:** biobanking, biospecimens, decentralized biobanking, precision medicine, blockchain technology, non-fungible tokens, NFTs, patient empowerment, futures methods, backcasting, futures wheel, visioning, participatory research, learning health system

## Abstract

**Background:**

Biobank privacy policies remove identifiers from donated specimens, siloing patients, discounting multimodal data, and hindering precision medicine. Decentralized biobanking is a new paradigm that unlocks value by uniting patients, specimens, scientists, and physicians in a blockchain-backed platform with robust incentives, governance, and ethical oversight. Informed by a real-world pilot, this mixed methods futures study explores how we advance decentralized biobanking from theory to practice.

**Objective:**

This study aimed to define the implementation strategy, synthesize pilot experiences into future vision, and highlight the implications and potential roadblocks.

**Methods:**

We applied backcasting from 2021 to 2024 through ethnography, alignment exercises, surveys, interviews, site visits, and futures workshops to map biospecimen supply chains and define principles for decentralized biobanking, using a breast cancer biobank for prototyping and software development. A decentralized biobanking app was piloted to engage breast cancer biobank members in participatory visioning. Thematic analysis of pilot experiences revealed a technology-enabled future vision. We systematically analyzed the pilot event via a Futures Wheel, organizing participant quotes as first-order effects, indirect effects, and anticipated implications.

**Results:**

Backcasting unveiled a pathway for designing an initial app for patients to track their biospecimens within institutional databases. We defined the “rails, rules, and tools” for a long-term, effective, and structurally just Biomediverse. Pilot enrollment was robust, and concurrent biobank enrollment was increased. Qualitative themes revealed impact on dignity, recognition, understanding, belonging, ownership, and empowerment. A vision for the future emerged from user journeys: “From ‘Lab Rat’ to Research Partner,” vividly depicted as a path transitioning from sterile graveyard to flourishing community garden. Primary themes were matched to first-order effects, indirect effects, and future implications, culminating in gratitude and unity, network effects reinforced by reciprocity, as well as potential for compensation and precision medicine.

**Conclusions:**

Reconnecting patients with their donated biospecimens via decentralized biobanking apps unlocks value for patients and aligns incentives across the Biomediverse. We illuminate the future person-centered biomedical data economy and put forward the goal of enabling all US biospecimen donors with decentralized biobanking by 2030.

## Introduction

Biobanks collect biospecimens, like tissue and blood leftover from clinical procedures, for biomedical research. To protect privacy, patient names are removed before specimens are accessed by scientists. Consequently, patients rarely learn about personal contributions to science, and scientists are prohibited or deterred from communication with donors under the terms of use [1]. However, eliminating patients’ connections to their specimens leads to ambiguous ownership, misaligned incentives, and missed opportunities for precision medicine. Manual, ad hoc collection, and siloed storage encourage hoarding and limit collaboration between public and private sectors [2-4]. Under this model, patients do not benefit from research on their specimens, even if it could save their lives, and most donated specimens are frozen or discarded. Current deidentification practices and broad informed consent further exacerbate these challenges, creating incentive misalignments and disconnecting individuals from downstream research activities.

**Figure 1.**
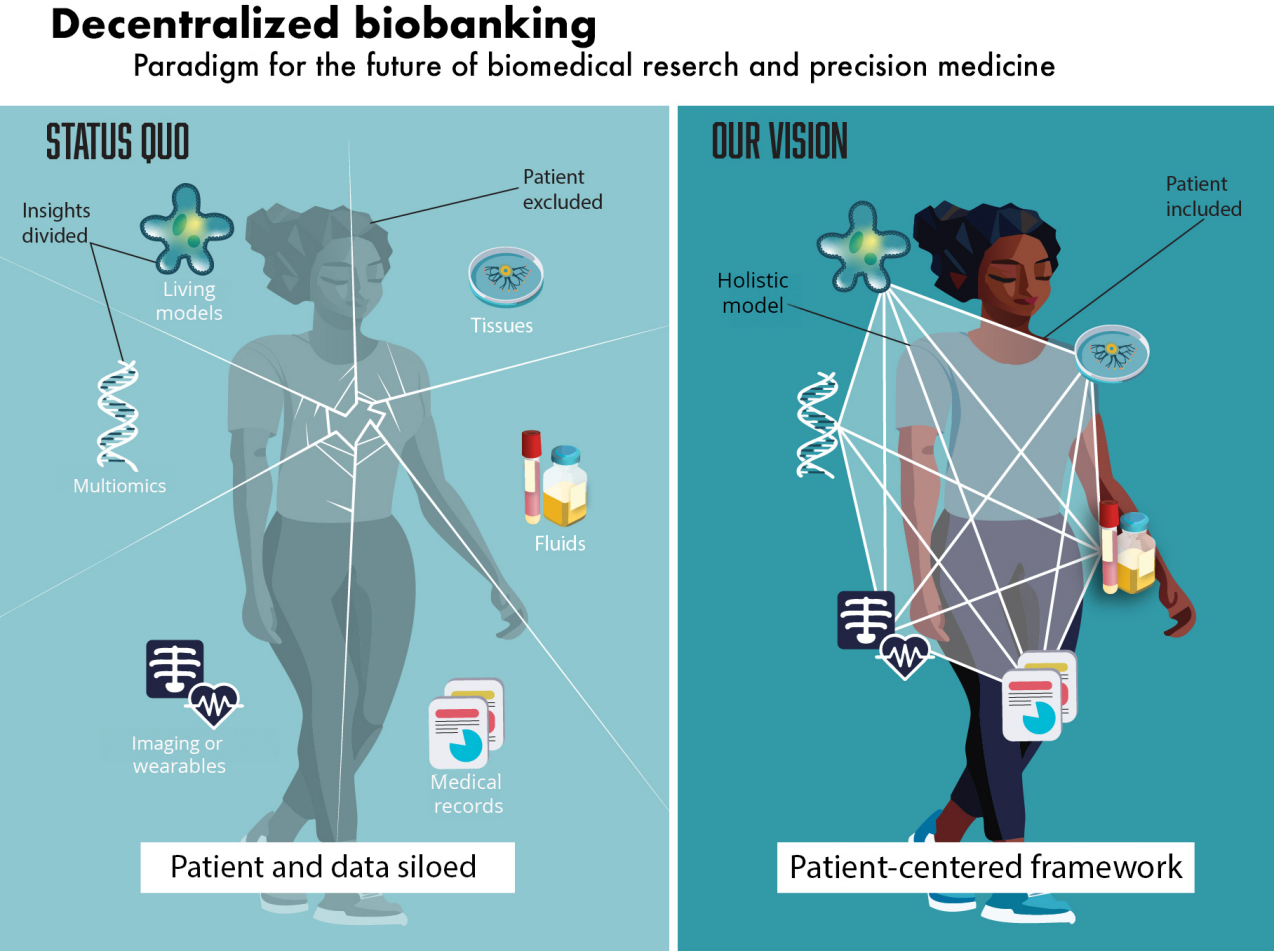
Left: current ethical frameworks and health systems silo patients from data derived from their biospecimens; right: vision for a patient-centered data framework that interconnects individuals with their biospecimen-derived data, unlocking opportunities to collaborate with a diverse ecosystem of physicians and scientists. Refer to detailed data typologies in Table S1 in Multimedia Appendix 1.

Although commonly viewed simply as clinical samples, biospecimens represent a unique opportunity to construct disease patterns and longitudinal timelines when integrated with patient demographics, clinical outcomes, or lifestyle factors. Furthermore, linking biospecimens to other datasets, such as genomic, proteomic, and pharmacogenomic data, can significantly enhance our understanding of how biological and environmental factors interact to influence disease initiation and progression. However, in the current paradigm, biospecimens and their associated data are siloed from patients and from each other, severely limiting harmonization and translational impact (Figure 1, left panel).

Decentralized biobanking represents a novel paradigm for biomedical research and precision medicine that centers patients as principals in a personalized health data architecture (Figure 1, right panel). Synthesizing bioethics, biobanking, and blockchain, our approach implements user-friendly software applications and blockchain-backed infrastructure to unlock value while meeting user needs across health care and biomedical research contexts. Decentralized biobanking (de-bi) emerges from the democratic ethos, transparency, tokenization (ie, the process of creating a digital asset or “token” that represents a physical item or right—in this case, a biospecimen or its associated data), and trust affordances of blockchain technology. Our “digital twin” ecosystem—a system in which physical entities, such as biospecimens or individuals, are represented by secure digital counterparts—represents entities as non-fungible tokens (NFTs), immutable, provably unique cryptographic assets recorded on the blockchain.

Blockchain technology supports decentralized biobanking by enabling secure, immutable tracking of biospecimens and associated data across multiple institutions. It facilitates transparent and tamper-proof management of patient consent, ensures traceability throughout the biospecimen life cycle, and enhances data security through decentralized storage and cryptographic protection. Together, these features support patient empowerment, transparency, and trust. A decentralized biobanking ecosystem is designed to be privacy-friendly, flexible, composable with legacy systems, and incentive-aligning with embedded potential for global impact [5].

As illustrated in Figure 1 (right panel), our vision positions patients as central stakeholders who are interconnected within an ethically governed sociotechnical infrastructure. Rather than being isolated and disconnected, patients, biospecimens, and data form an integrated “digital twin” framework that enables peer-to-peer connections, aligns stakeholder incentives, and mobilizes patient interests to the forefront. In this envisioned “Biomediverse”—a future-state biomedical ecosystem where patients, scientists, institutions, and data systems are interconnected through decentralized infrastructure—continuity of care for patients extends to continuity of care for their data, empowering individuals with knowledge about and control over their biospecimens throughout the research life cycle.

**Figure 2.**
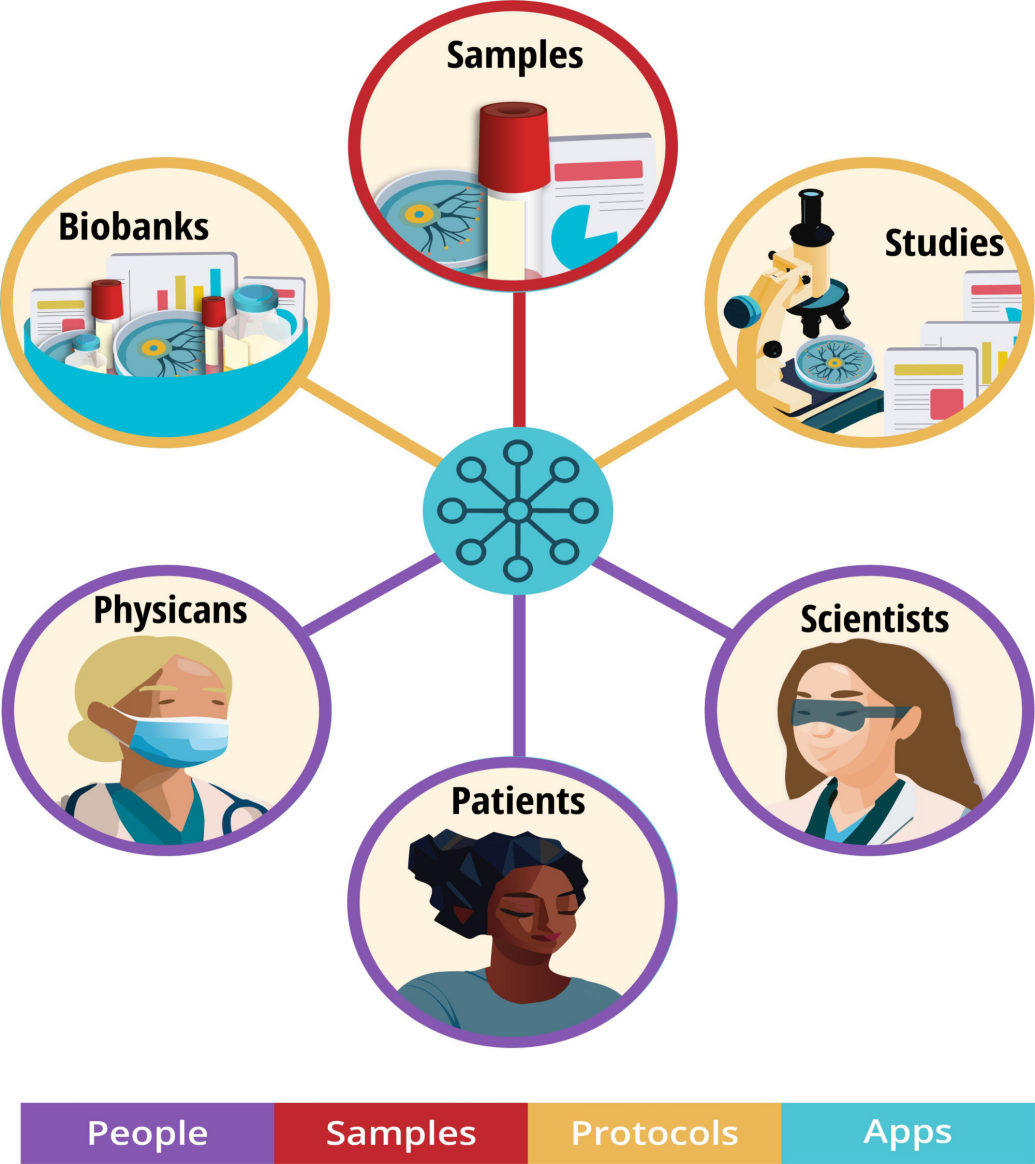
Biomedical metaverse “biomediverse” schematic: decentralized biobanking ecosystem of people, studies, and samples.

Achieving this vision requires engaging patients as principals with enduring interests in their specimens to disintermediate supply chains, facilitate linkage of currently siloed data, and accelerate precision medicine. This decentralized biobanking ecosystem is presented schematically as a digital twin framework in the Results section (Figure 2).

Patients with cancer, in particular, have benefited significantly from biospecimen-based translational research, leading to marked improvements in clinical outcomes over recent decades. Despite this progress, patients with cancer remain largely disconnected from the fate of their donated biospecimens and rarely receive potentially life-changing research findings derived from their samples. By reconnecting patients with cancer and scientists within the decentralized biobanking framework, we aim to remove critical barriers to precision oncology, facilitating tailored interventions.

We apply backcasting—a strategic planning method that begins with a desired future state and works backward to identify the steps necessary to achieve it—to advance decentralized biobanking, seeking a path forward that overcomes tension between utility, value, and efficiency of biospecimens while upholding donors’ rights to respect, privacy, and benefit sharing [6,7]. This mixed methods futures study identifies first steps for reducing de-bi to practice by working backward from our proposed ideal for a biomedical metaverse, or “Biomediverse.” We define the Biomediverse as a future-state biomedical ecosystem in which patients, scientists, institutions, and data systems are interconnected through decentralized, ethically governed infrastructure that promotes transparency, aligns incentives, and enables collaborative discovery. We define the initial implementation strategy, informed by technical and qualitative experiences from the first real-world de-bi pilot [8,9]. We review participant quotes to assess our proposed approach, inspired by participatory visioning and aided by a Futures Wheel to identify insights, barriers, and next steps.

## Methods

### Overview and Timeline

This study used multiple complementary methodological approaches conducted between 2021 and 2024. To define a clear roadmap toward the envisioned decentralized biobanking ecosystem, we first applied backcasting to identify foundational long-term investments and strategic interventions. This was followed by ethnographic research and stakeholder engagement to gain contextual understanding and identify viable points of intervention. Subsequently, iterative user-centered design and blockchain-based software development were conducted to create and validate a biospecimen-tracking app. This app was then evaluated through a real-world pilot study with biobank participants. Finally, qualitative analyses—including participatory visioning exercises and a Futures Wheel analysis—were conducted to synthesize and systematically explore user experiences, emergent insights, and potential future implications. Table 1 summarizes the sequence and timeline of these methodological components.

**Table 1.** Overview of research methodology and timeline.

2021	2022	2023	2024
Problem framing Ideation Visioning Stakeholder meetings and preliminary feedback Use case selection Initial prototype	Alignment exercises with key stakeholder types Futures Workshop Site visits Surveys ×2 Stakeholder interviews Design Workshops More prototypes	Develop and refine apps Real-world pilot for breast cancer biobank with survey Cognitive walkthroughs and app onboarding Stakeholder Presentations Participatory visioning and community engagement More interviews	Feasibility analysis: technical, operational, regulatory, and economic Data synthesis for Futures Wheel Development of new technologies Real-world pilot for translational research with survey Comprehensive roadmap and policy framework

### Backcasting Approach

We applied backcasting, a strategic planning method that begins by defining a desired future state and then systematically works backward to identify the steps and interventions required to achieve it. This approach was selected to explicitly clarify long-term goals for decentralized biobanking and avoid incremental solutions constrained by current biobanking limitations. Our team defined an ideal “Biomediverse” as a future-state biomedical metaverse comprising an ecosystem in which biospecimen donors, researchers, and institutions are interconnected through a decentralized, ethically governed, sociotechnical infrastructure that promotes transparency, aligns incentives, and enables collaborative discovery. Then, we worked backward from this ideal state, identifying technical, ethical, and operational milestones required to realize this vision, and guiding our strategic roadmap and methodological choices throughout the project.

### Baseline Assessment (Ethnography and Stakeholder Input)

We conducted ethnographic research, supplemented by stakeholder engagement, to map current biospecimen supply chains, identify existing incentives, and clarify barriers within the biobanking ecosystem. Key stakeholders, including patients, physicians, biobankers, scientists, institutional review board (IRB) members, institutional leadership, and industry professionals, participated in structured alignment exercises and futures-design workshops (Table S2 in Multimedia Appendix 1) [10-12]. These engagements, complemented by site visits, structured interviews, and surveys, provided detailed contextual insights into the prevailing state of biobanking practices and stakeholder concerns. Through this baseline assessment, we identified specific, actionable problems and opportunities, prioritizing value propositions with a focus on viable, high-impact solutions with near-term utility to inform subsequent app development and pilot implementation.

### Real-World Biobank Setting

Initial research and development occurred in the setting of one of the world’s largest dedicated breast cancer biosample collections (STUDY19060196 refers to Breast Disease Research Repository [BDRR]: Tissue and Bodily Fluid and Medical Information Acquisition Protocol). From 1995 to 2023, roughly 11,000 patients consented to donate, from which 4000 unique donors contributed approximately 61,000 specimens. Nearly 10% (5663/60,792) of the inventory was distributed for research, and roughly 90% (55,130/60,792) remained in long-term frozen storage. The BDRR is a hub-and-spoke model biobank nested within an institutional biobanking platform at a US NCI Comprehensive Cancer Center and features a flagship living biobank of approximately 300 patient-derived breast cancer organoids. Organoids are 3D tumor models grown in the laboratory, a culturally, clinically, and commercially significant biospecimen use case exemplifying the urgency and potential impact of decentralized biobanking [13].

### App Development and Blockchain Integration

#### Defining the Initial Application

Initial app development focused on creating a patient-friendly digital interface enabling biospecimen donors to track their donations throughout the biomedical research life cycle. Leveraging insights from baseline assessments, we prioritized intuitive design and minimal onboarding friction, inspired by familiar digital experiences such as online banking applications, regularly used by 89% (25/28) of patients surveyed. We hypothesized that such a technology could provide personalized feedback while remaining compliant with deidentification, compatible with established workflows, and composable with ongoing collection protocols. Successfully demonstrating these features would yield a scalable mechanism for providing passive transparency for donors, without requiring active participation from physicians, biobanks, or scientists. Hence, specimen tracking may minimize friction for onboarding while simultaneously building trust and generating opportunities to unlock value for dense atomic networks of patients and scientists with shared assets and interests [14]. Key milestones of the app development and integration process are outlined in Textbox 1.

Textbox 1. **Key milestones for patient-centered biospecimen tracking.**

- Design patient-friendly biosample tracking app
- Develop and integrate app with institutional biobank
- Deploy app for real-world biobank pilot use case
- Demonstrate potential impact and value of patient engagement

#### Blockchain Integration and NFT Implementation

We introduce our NFT digital twin ecosystem**,** which deploys a suite of smart contracts on a public, permissionless blockchain network (ie, Ethereum) used to create nonfungible tokens as digital representations of stakeholders and assets, as well as research and biobanking protocols, mirroring real-world biospecimen research networks. The benefits of blockchain technology include trust through embedded transparency and immutability, as well as enhanced interoperability across institutions. The NFT digital twin ecosystem allows for symbolic representation of complex entities, activities, and data in a simplified, widely accessible format that can grow in complexity and in utility over time. This approach ensures traceability, establishes provenance, and enables ethical stewardship of donated specimens [13,15].

For this specific pilot, a nontransferable ERC-721 NFT, also referred to as a “soul-bound token” [16], was minted as a representation of each donor’s unique and permanent connection to their donated biospecimen. These “Biowallet NFTs” were minted for participating donors as both a “token of appreciation” for their contributions and a functional access control mechanism for unlocking their in-app Biowallet and viewing additional details about their own samples. After a series of prototypes, we developed and deployed a de-bi mobile app for Android (Google) and iOS (Apple Inc) [17]. Additional details regarding system architecture, technical implementation, and coordinated processes for this pilot are reported elsewhere.

#### Privacy-by-Design Implementation

We apply principles of data minimization as each NFT is identified via a unique, cryptographically hashed ID for facilitating secure data linkage and reliable access and permissions management while maintaining any identifiable information and linkages to patients off-chain on the secure institutional repositories where they are currently maintained. The blockchain and digital assets serve simply as a decentralized index or reference layer, reducing the risk of exposing any sensitive data. Our backend server integrates with these institutional systems to automatically reflect notable activities on-chain, maintaining provenance between the NFTs and their real-world counterparts. This approach embeds transparency into the biospecimen research life cycle while protecting patient privacy and remaining compliant with established deidentification protocols [18-21]. The app was tested and refined with potential users from October 2022 to January 2023 (QRC 3958).

### Pilot Study

Biobank members were recruited from February 16 to April 30, 2023 for a real-world pilot study to evaluate the biospecimen-tracking app (IRB 22020035). Approximately 600 invitations to download and test the app were distributed to eligible biobank members. Data collection, regulatory considerations, as well as technical and operational feasibility, are reported in-depth elsewhere. Refer to Table S3 in Multimedia Appendix 1 for demographics.

### Data Sources and Qualitative Analysis

Qualitative data were collected from multiple sources, including participatory futures exercises, design workshops, pilot study correspondence, cognitive walkthroughs, immersive engagement events, and app onboarding sessions (both virtual and in-person). Data included verbatim quotes from participants, written reflections, video transcripts, and direct observational notes. In total, 4 independent research team members conducted thematic analysis through an initial round of open coding, followed by iterative refinement and discussion to reach consensus on key themes. This systematic approach enhanced the reliability and validity of identified themes related to participant values, perceptions, and experiences with decentralized biobanking.

### Participatory Visioning

Mixed methods research, community engagement, and pilot testing provided the foundation for participatory visioning. Over several years, we engaged extensively with participants as they considered future prospects of decentralized biobanking, rediscovered their specimens via the de-bi app, and grappled with present implementation versus future potential. Pilot participants engaged in futures-focused exercises, including letter-writing, imagining alternative futures, and reflecting upon how de-bi might impact relationships with their own body, biomedical research, and the healing process [22]. Through thematic synthesis of participant quotes integrated with quantitative data, we developed a vision of research transformation coalesced from app users’ lived experiences [23]. Narrative synthesis was graphically depicted and refined iteratively through a series of reflections. Representative quotes were mapped onto the emergent vision of an evolutionary model.

### Futures Wheel Analysis

The pilot was selected as the key event and placed at the center of a Futures Wheel, a structured visual methodology used to systematically explore and map out direct and indirect consequences of an event or decision, helping to identify potential long-term implications. Direct effects were mapped from representative quotes from primary themes of participant feedback during app engagement and onboarding. Indirect effects were visualized by dissecting higher-order lived experiences, as described by participants. Finally, potential implications of decentralized biobanking, as articulated by the pilot participants, were organized as offshoots of the indirect effects, suggesting an array of potential positive and negative feedback loops [24]. Representative quotes illustrating each level of effect (direct, indirect, and implications) were systematically organized and visually mapped onto the Futures Wheel to depict the connections between the pilot experience and anticipated future outcomes.

### Ethical Considerations

Mixed methods research herein was enabled by several IRB-approved protocols and a quality improvement protocol, as listed below. The participants in each of the protocols below provided informed consent or waived informed consent, in accordance with the respective protocols. Conflicts of interest disclosures were included in all consent procedures and reiterated as appropriate at each virtual or in-person encounter. All presented data are deidentified. Participants were not compensated. IRB00019273 was obtained for NFTs for ethical, efficient, and effective use of biosamples (Johns Hopkins University). STUDY22020035 refers to Decentralized Biobanking “de-bi” for exploring patients' interests in feedback, education, follow-up, engagement, and tokens of appreciation regarding biobank donation via mobile and web applications (University of Pittsburgh). STUDY19060196 refers to BDRR for tissue and bodily fluid and medical information acquisition protocol (04-162; Hillman Cancer Center). QRC 3958 refers to patient-facing Biobank Platform Development QI Proposal for Beckwith Award – Breast Cancer Supply Chain Analysis, Biobank Token Model Development, and Initial Pre-Pilot Testing with UPMC Patients (University of Pittsburgh Medical Center).

## Results

### Pilot Study Results

In total, 1080 participants enrolled, representing nearly 10% (930/9750) of eligible biobank members. Out of the approximately 600 app invites distributed, 405 participants downloaded the app and completed onboarding during the 10-week pilot. Collectively, 272 participants were reconnected with 3904 biospecimens, with a mean of 14.5 specimens per donor (range 1‐84). Of these, on average, 1.4 (range 0-9) specimens per donor were distributed for research. Notably, biobank enrollments increased by 65% (from 158 enrollments in 2022 to 261 enrollments in 2023; *P*<.001) during the pilot recruitment period. No participants withdrew from the biobank during or for 1 year following the pilot (*P*<.001).

### App Features and User Engagement

**Figure 3.**
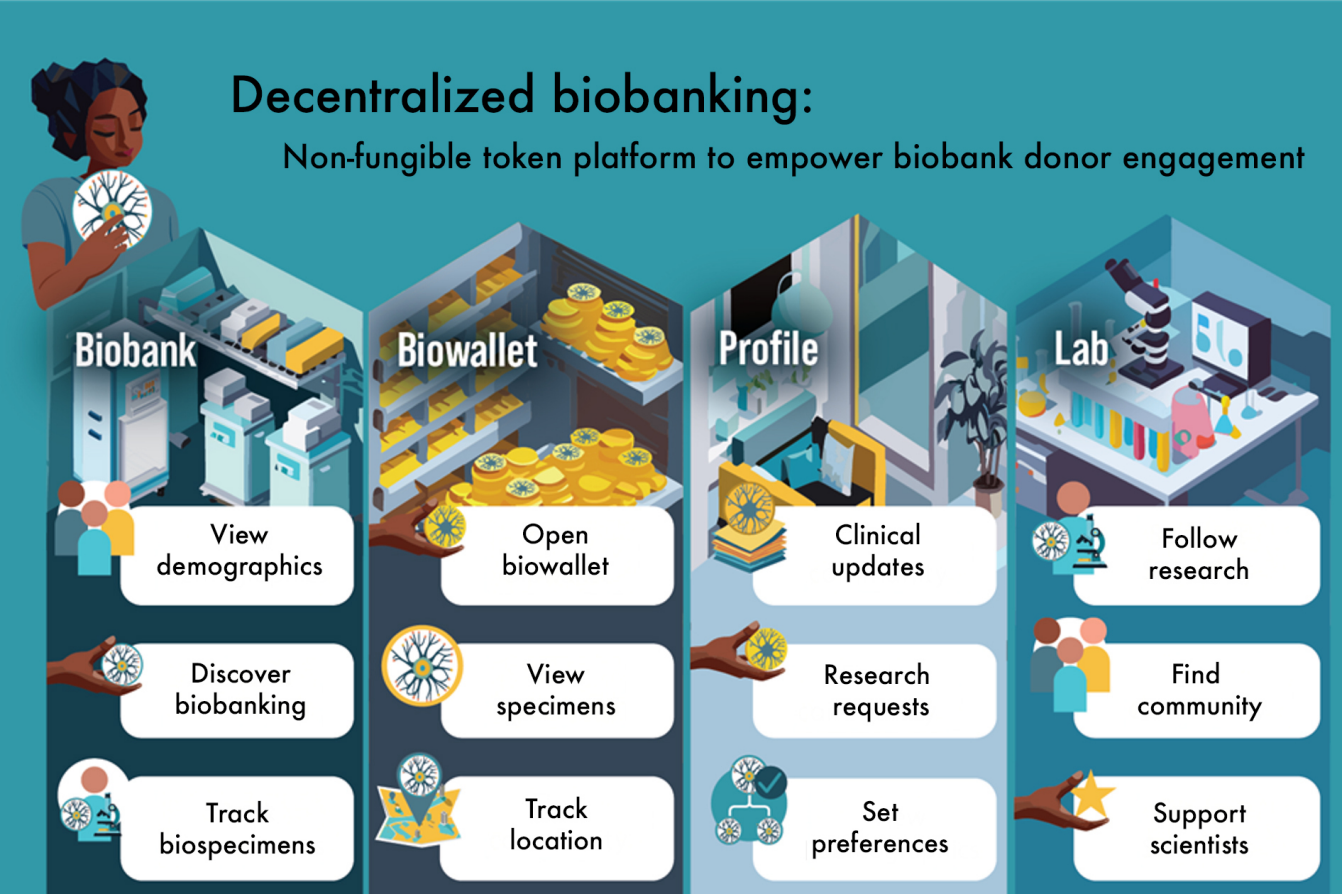
Decentralized biobanking platform for biospecimen donors: technology solution overview and user journey map.

In total, 4 contexts and corresponding features were introduced, providing accessible personal-level and community-level specimen tracking (Figure 3) [17]. We illustrate (1) “Biobank” where donors could learn about biobanking and track the entire collection; (2) “Biowallet” where donors could view and track the status of personal specimens; (3) “Profile” where donors demonstrate the ability to share clinical data, annotate specimens, and set research preferences; and (4) “Lab” where donors could follow research activities enabled by their specimens and engage with personalized communities. Detailed app user journeys and NFT components are reported in studies by Sanchez et al [18] and Gorijavolu et al (unpublished data, 2025).

### Participatory Visioning: From Scavenger Graveyard to Garden of Empowerment

#### Thematic Overview

Rich visions were constructed from in-depth narratives (n=10), drawing primarily from 6 transcribed videos and 6 writing samples. These were thematically reinforced by written feedback from design workshops, direct quotes from over 100 unsolicited emails from the pilot, and immersive community engagement. Overall, participants were enthusiastic about the chance to track their biospecimens, seeing the platform as a source of personal meaning and deepened knowledge of the biomedical research process.

#### Qualitative Findings: Direct Participant Experiences

Participants described their previous experiences with traditional biobanking as disconnected and impersonal, with limited understanding of the fate of their biospecimens:

*I was originally diagnosed with breast cancer, I underwent surgery and samples were taken, and I know I signed a consent right before a procedure occurred, but I never thought about ‘what’s happening with the stuff that people are carrying away from me?’ I thought of it as, ‘This information is going to be used for my medical team to make decisions about this treatment or that treatment.’ But I hadn’t thought about ‘What else happens with my cells?’*

The consenting process for biosample donation was perceived by some as a loss of bodily autonomy, exemplified by a participant reflecting on her radical mastectomy experience:

*The very moment I gave my consent…my connection to my own tissue was about to come to an abrupt end in the cold sterility of the surgical suite. Whatever fruit those cells might yield in the lab was destined to be… remote to me.*

By contrast, through engagement with the decentralized biobanking pilot (de-bi), she expressed a renewed sense of human “dignity” and bodily integrity:

*One might say that de-bi has provided for the re-membering of my bodily integrity…From radical mastectomy to radical restoration.* [25]

The deeply personal nature of breast cancer surgery underscored participants’ interest in following their tissues, addressing the unmet need for “recognition,” as illustrated by the statement:

*I want to be recognized, the acknowledgment of like, yes, that’s my sample.*

The pilot platform facilitated a deeper “understanding,” as in:

*...with this special app…I can essentially peek in and see what’s happening.*

Participants experienced an awakening of awareness about biobanking, exemplified by a participant’s vision:

*Our cells…are sitting somewhere, like Sleeping Beauty, waiting to be kissed...But…instead of a kiss, I get an app…*

The user experience of the de-bi app also engendered “belonging.” For example:

*You’re not out there alone. You can go onto this app and see that there are other people who have this kind of tumor or diagnosis.*

Participants made comments like:

*“I can’t wait to see what’s being done with my samples!”* (original emphasis and punctuation)

By tracking corporal contributions to science, we portended the sense of “restored ownership” of the body, evoking a digital repatriation of dehumanized byproducts [16]. Finally, participants indicated “empowerment,” emphasized by one who said:

*With this app, everybody becomes a colleague…. Because they know, I’ve got these cells, I’ve got these samples, and it’s my cells, it’s me, who’s actually pushing the science forward…*

Reflecting, she noted:

*It’s at those moments when you realize that this is a collaborative effort, right? We, as patients, are giving as good as we’re getting.* [26]

The qualitative feedback underscored the transformative potential of decentralized biobanking, illuminating direct effects on “dignity, recognition, understanding, belonging, ownership, and empowerment” (Table S4 in Multimedia Appendix 1).

**Figure 4.**
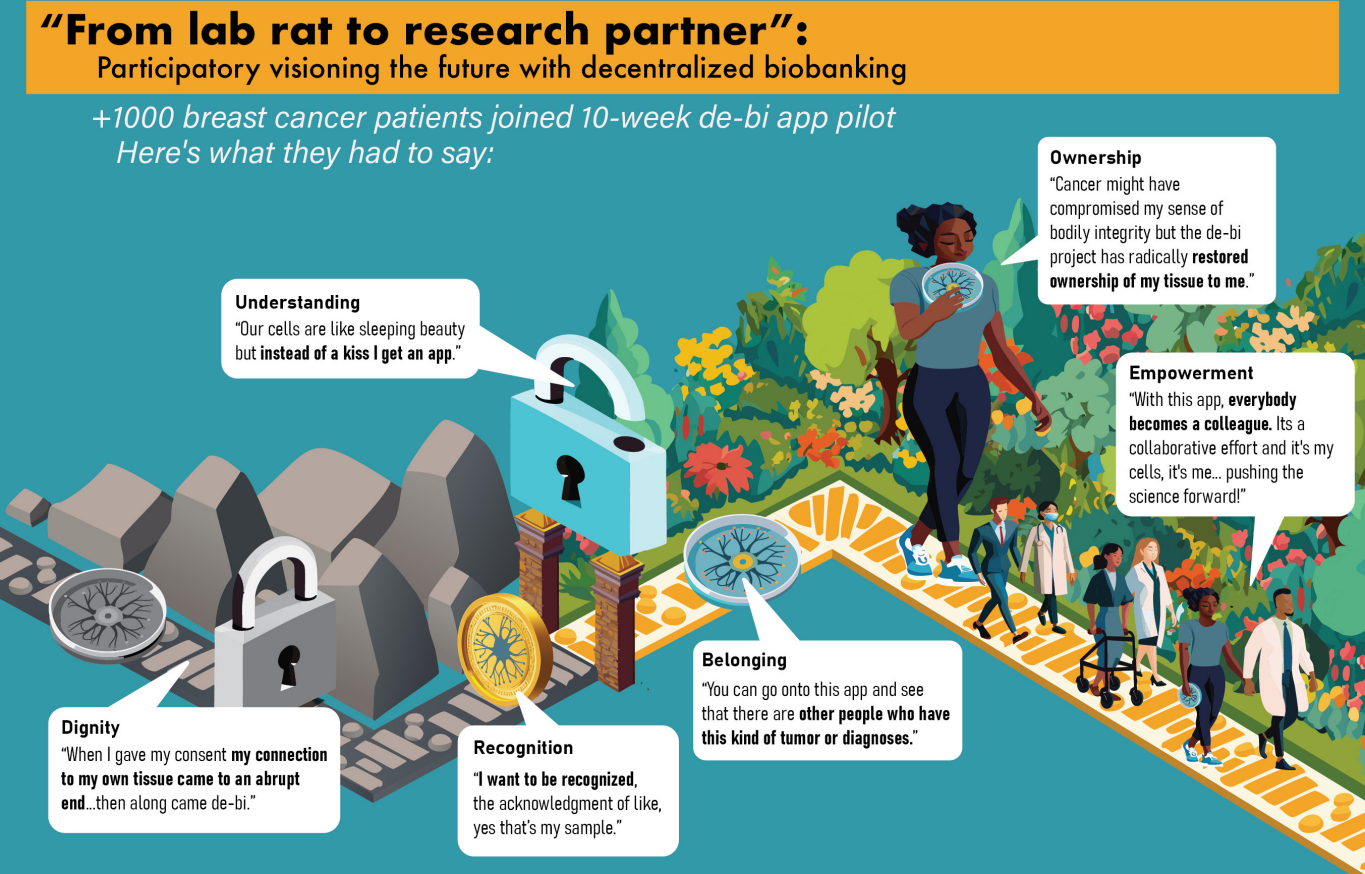
“From lab rat to research partner:” decentralized biobanking transformation.

Participatory engagement yielded a future vision in which digital infrastructure embeds transparency, unlocks value, and fosters multistakeholder collaboration. The direct effects of the app, as expressed by pilot participants, were organized in a cumulative process. The overarching narrative was synthesized into vivid imagery, representing the transition from a sterile graveyard of biospecimen donation to a vibrant community garden for research collaboration (Figure 4). Representative quotes were mapped along the route, illustrating the transformation, summarized by one participant:

*I feel I am moving from the role of ‘lab rat’ to research partner.*

This phrase emerged directly from participant language and reflects a shift in how patients perceive their role—from passive, deidentified “subjects” to active collaborators in research—and carries metaphorical and narrative power in conveying the profound transformation experienced by pilot participants.

### Qualitative Findings: Indirect Effects and Broader Implications (Futures Wheel)

**Figure 5.**
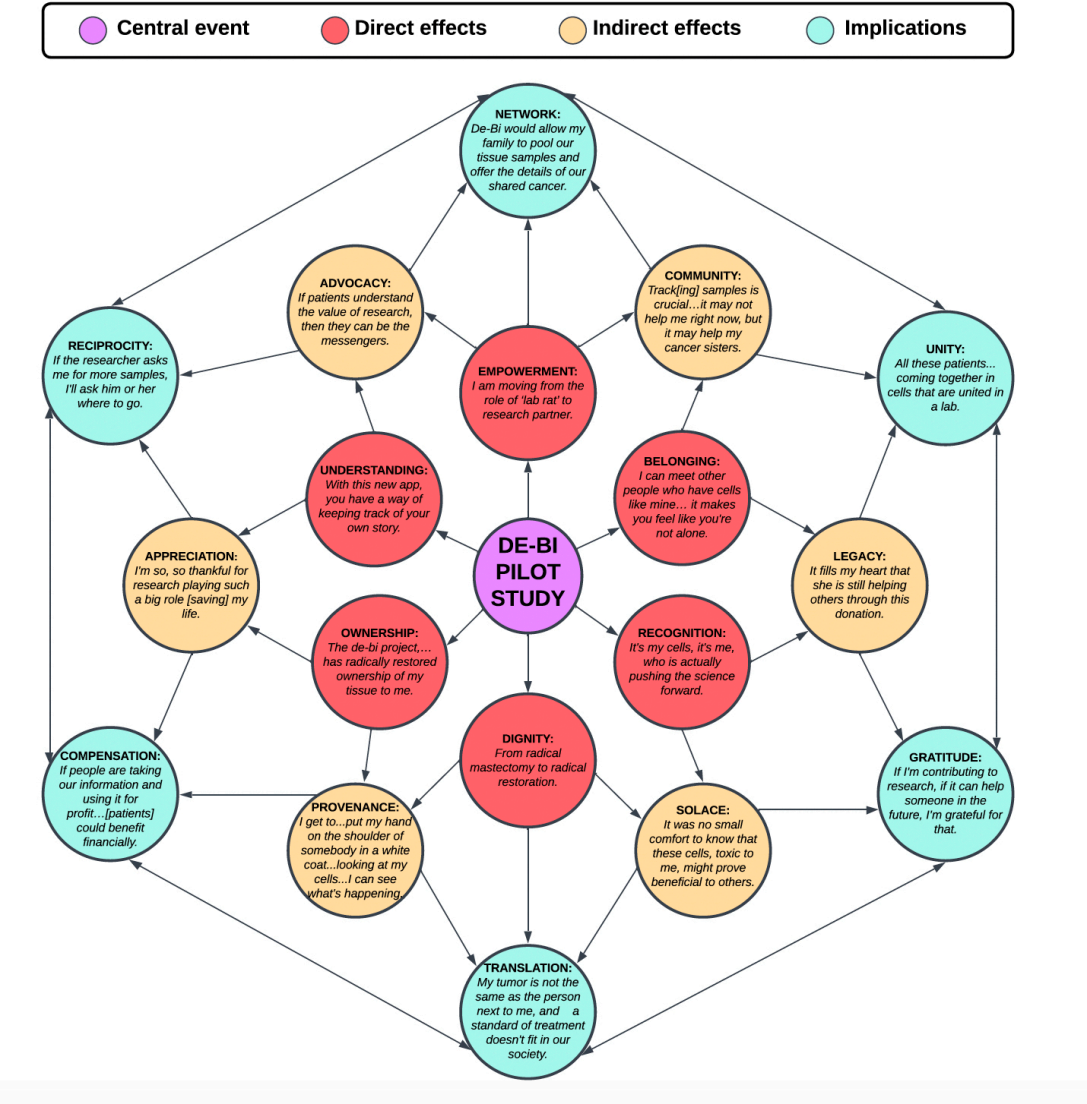
Participant-driven futures wheel: direct effects, indirect effects, and future implications of decentralized biobanking. de-bi: decentralized biobanking.

Building on direct effects, we mapped indirect effects of the pilot onto a Futures Wheel, with themes of “appreciation,” “advocacy,” “community,” “legacy,” “solace,” and “provenance.” Future implications for “gratitude,” “unity,” “network,” “reciprocity,” “compensation,” and “translation,” further emerged, illustrating potential feedback loops that may accelerate or stymie adoption (Figure 5) [27].

#### Indirect Effects

Pilot participants emphasized “appreciation” for research, attributing their survival to contributions of previous donors and scientific achievements. For example:

*Some might say that I’m lucky…but I’m very aware it’s not just luck. My successful treatment with Herceptin was the product of many years of medical research. And that research was made possible by women like you and like me who donated their tissue samples.*

Participants illustrated how seeing the impact of personal contributions would promote a positive feedback loop via “advocacy”:

*If patients understand the value of research, then they can be the messengers. They can be the advocates for all kinds of research, not just research that affects them individually…*

The pilot reinforced a sense of “community,” especially through the virtual laboratory environment, where participants were able to track their specimens alongside those from individuals with a similar location, study, diagnosis, or investigator. Explaining the value of our approach, one patient said:

*It may not help me right now, but it might help my cancer sisters*.

Intimacy was deepened through the interpersonal exchanges of 3D human matter, highlighting a profound sense of personal and collective identity tied to biological research contributions. “Solace” emerged as a silver lining, illustrated by one patient who wrote:

*It was no small comfort to know that these cells, toxic to me, might prove beneficial to others.*

Tracking samples was especially important for some facing metastatic disease, who may be reassured, achieving immortality through seeing their “*cells are still there to contribute to scientific discoveries,*” even after death. An enduring “legacy” was forged for individuals and their loved ones, whereby the ongoing impact of research donations seemed to meaningfully extend lifespan. The widow of one donor emailed:

*It fills my heart that she is still helping others through this donation…it would be a gift to let [our children] know their mom is still helping others from the cross she had to bear.*

Digital representations of biospecimens were viewed as concrete cellular subcomponents arising from the embodied self, laden with psychosocial, emotional, and clinical significance. Motifs of materiality and “provenance” were central, powerfully articulated through one participant’s comparison of herself to the narrative of Henrietta Lacks:

*There was no connection between Ms. Lacks and all the treatments that have ensued. But this way, it’s as though I get to step into Henrietta Lacks’ body, and walk into a lab somewhere, and put my hand on the shoulder of somebody in a white coat, bent over a dish, looking at my cells, trying to figure something out…and I can see what’s happening.*

#### Implications

The app fosters “gratitude” by making users feel valued and acknowledged, and allowing them to express the same for fellow donors and scientists. For example, one noted:

*I am so, so thankful for the role of research in [saving] my life*.

Another stated:

*If I’m contributing to any kind of research, if it can help someone else in the future, I’m grateful for that.*

The donation of tumor tissue was salient, as the very object that harmed the donor was converted into a tool for benefiting others.

Participants internalized recognition of their role in research as a way of being part of something larger than themselves, finding “unity” in the global effort to “outsmart cancer.*”* For example, one notes:

*Rarely does science change with one patient. But when you have ten, and ten to the second power, ten to the third power… all coming together in cells that are united in a lab, that’s when science changes.*

Another stated:

*We are all in this together. The de-bi project is an important step toward maximizing research benefits for everybody.*

One explained:

*The app would be very beneficial …because what I have is rare, so there might be a…chance that I could find somebody else out there who is…going through the same thing.*

The potential to augment patients’ support systems by combining the social value of togetherness with opportunities for personalized learning was emphasized [25].

The application illuminated a hidden “network” of connections within the biobanking ecosystem, enabling future potential for linkage of similar specimens, donors, and data across institutions. Participants recognized the potential for digital infrastructures to build trust and promote collaboration. For example, one wrote*:*

*My family is at high risk for breast cancer yet negative for all currently known breast cancer genes. De-Bi would allow my family to pool our tissue samples and offer the history and details of our shared cancer.*

She highlights how decentralized biobanking may help answer outstanding questions, unlocking “potential discovery of a new breast cancer gene by studying our tissues.*”*

Others highlighted how feedback loops may spur “reciprocity,” supporting serial specimen donations, as in:

*If the researcher asks me for more samples, I’ll ask him or her where to go and I will be there.*

Participants noted how the app might help drive usage of frozen specimens, with both personal and prosocial benefits in mind. For example:

*As more and more people learn about the de-bi app, you as a patient [can use the platform] to let researchers know that your tissue is out there for them to use in research*. [25]

This sense of agency may inspire participants to contribute more actively and suggests potential to amplify recruitment, retention, and engagement. For some, feedback about their personal contributions is its own reward, as in:

*I am more interested in being able to see how my specimens are used in research. If they can help at least one person, it will do more good for me than money possibly could*.

By contrast, several participants envisioned how de-bi might allow patients to share profits derived from their specimens, with supply chain transparency enabling new economic models for just “compensation.” Patients were most interested in accessing commercial gains as redress for the financial toxicity of cancer, especially among young adults whose careers may have been cut short by their disease. Meanwhile, some were concerned about “how to prevent misuse and financial gains by others,” highlighting how perceived lack of respect for patient sovereignty may erode trust. Transparency regarding commercialization represents a potential source of negative feedback that may challenge institutional adoption or credibility among users, poignantly captured by one participant who said*:*

*It pisses me off that they make so much money off of us, but I don’t want their money– I just want a cure, I just want to be here for my kids.*

Ultimately, participants recognized the potential for de-bi to facilitate personalized medicine, informed by firsthand knowledge that “everybody’s cancer is different…and what works for one person might not work for another*…*” and inspired by how bridging bench and bedside “will enable the correct treatment to be used*.*” One participant in her 20s was attuned to the need for direct “translation,” stating:

*I know that my future [survival] depends on both advocacy and research and this app has the potential to be a remarkable tool in my journey.*

Similarly, participants discussed how de-bi might democratize precision medicine, as in:

*Everybody should be able to have this research done because my tumor is not the same as the person next to me…there are multiple drugs, multiple tumors, so why not find out what works?*

### Integrated Framework and Core Elements

Taken together, the results previously above—spanning pilot outcomes, participant experiences, and perceived implications—highlight core elements of a decentralized biobanking ecosystem. Decentralized biobanking presents the “rails, rules, and tools,” of a structurally just Biomediverse wherein all people, protocols, and biological products are connected via symmetrical digital and moral infrastructure (Table 2). Our NFT digital twin ecosystem combines human-centered architecture, an ethically governed marketplace, and composable software applications that synergistically advance the universal mission of individual health and collective human flourishing.

**Table 2.** Decentralized biobanking: components and features.

Element	Component	Features
Rails	Human-centered architecture	Digital infrastructure maps and maintains relational integrity for people, samples, and studies via a global digital twin overlay network.
Rules	Ethically governed marketplace	Informed consensus aligns incentives and optimizes efficiency via market design powered by transparency, tokenization, and democracy.
Tools	Seamlessly integrated applications	Composable channels support long-term research engagement, optimized resource allocation, and timely translation of precision medicine.

## Discussion

### Big Picture

Biobanking systems are not designed to keep biospecimen donors informed; consequently, return of results is the exception, not the rule, and most biobanks are underused and insolvent. Decentralized biobanking reimagines biomedical research and precision medicine with a person-centered health data framework that aligns incentives for patients, physicians, and scientists. This futures study applies backcasting to define core, nonoverlapping principles of decentralized biobanking—summarized as “rails, rules, and tools”—for a Biomediverse that maximizes utility, value, and respect for biological assets. We mobilize theory into practice through design and deployment of a real-world pilot [9,18], demonstrating a technology solution for reconnecting donors to their biospecimens, enabling personalized feedback without compromising privacy protections [28].

Our framework provides transparent digital infrastructure and collaborative governance solutions to close gaps in current health systems that waste most biological assets and permit missed opportunities to save lives. Decentralized biobanking implements distributed shared ledgers, tokenization, and privacy-friendly innovations in a stepwise fashion, progressively unlocking cumulative layers of value from existing silos [29,30]. These technologies may help restore trust through advancing a shared state of truth in which the rights and interests of individuals and enterprises may be maximally aligned [31].

We engaged institutional leadership, regulatory specialists, and biobank stakeholders to identify specimen tracking as the essential first step for systemic change. Doing so allowed us to zero in on the “low-hanging fruit” to advance a technically straightforward and cost-effective solution, with potential to deliver outsized benefits while minimally burdening existing cultures, workflows, and infrastructure [11]. Participatory visioning demonstrated how a digital “*seat at the table*” for donors may catalyze a paradigm shift from “*lab rats*” to citizen scientists. As mentioned in the Qualitative Findings section, this phrase—drawn from participant language—captures a transformative perceived shift from passive “subject” to active research partner and reflects the narrative power of the transformation experienced by pilot participants [32]. The Futures Wheel illustrates how de-bi technology may represent an open platform with positive feedback loops and network effects, creating an avenue for future precision medicine and profit-sharing.

### Pilot Reflections

Nearly 10% (930/9750) of biobank members signed up to track their specimens in just 2 months, and there was a 65% increase in biobank enrollment during the pilot versus the same time in the previous year. Personalized specimen tracking positioned the application as a groundbreaking tool for research engagement with implications for health and wealth. For some, donated biospecimens exhibited a “phantom limb” phenomenon: gone, but never truly forgotten [33]. Compared with blood and urine samples, breast tissues exuded special meaning, possibly in relation to their structural role in reproduction and identity formation. Mapping direct and indirect effects revealed profound positive feedback loops that may enhance donor participation and data sharing through empowerment and reciprocity.

Our approach fosters belonging by connecting user communities with related tissues, diagnoses, or research investments, promoting long-term engagement by unlocking authentic relationships [34-36]. We also leveraged nonfungible tokens to facilitate implementation of patient rights and inclusion in the biomedical data economy, all while maintaining compliance with deidentification standards. By allowing individuals to share data and vote on research proposals, we suggested potential to accelerate research via patient-driven integration of clinical data. As participants engaged in annotating their specimens and following donations to specific protocols, they sensed implications for how their preferences and interests might direct specimen allocations in the future.

However, with enhanced engagement comes the need to define rights and guardrails to support patients and institutions, particularly regarding data ownership, control, and commercialization. If respect for donors’ agency does not meet expectations, negative feedback loops may emerge, potentially undermining trust and jeopardizing institutional reputations [37]. Disappointed participants may withdraw, prompting scientists to be fearful of repercussions from earnest disclosures regarding research limitations, funding, monetization, or other sensitive topics [38]. Thus, decentralized biobanking may reveal ethical dilemmas for the research enterprise, potentially sparking skepticism or resistance to adopting transparency. Careful design and communications are essential for patients and professionals alike and must provide clarity about current capabilities and governance of the platform, in addition to potential futures that our approach may spark.

Decentralized biobanking may generate an inclusion-advocacy flywheel. By providing a window into individual and group-level biobank activity, donors can be included in the research process, in turn promoting further involvement and creating new opportunities for longitudinal collaboration [39]. Analogous to quadratic funding—a resource allocation method that increases the impact of widely supported contributions by giving greater weight to projects backed by many individuals—decentralized biobanking has the potential to amplify patient voices and distribute power across the research ecosystem [40]. Thus, specimen tracking suggests potential for early return on investment via promoting research recruitment, retention, and engagement efforts. This flexible platform opens multiple possible benefits for the research enterprise, wherein 80% of clinical trials fail to meet enrollment targets, community engagement is a funding criterion, and linkage of serial specimens and multimodal data is essential to data quality and scientific impact [41].

Biospecimens contain numerous layers of key information specific to a patient’s tumor, but so many of these potentially game-changing biological clues remain sequestered within the research laboratories of academic institutions. By facilitating sample tracking and data sharing between scientists, patients, and their care teams, we will remove the veil that has separated these indispensable stakeholders for far too long. Subsequently, patients will be able to unlock the value of their own biospecimens in an unprecedented way. This network of transparency has the power to transform patient outcomes through the following: (1) discovery of novel cancer mutations and molecular subtypes, (2) accurate prediction of patient response to treatment, (3) identification of new drug targets within a specific cancer and, perhaps most importantly, (4) allowing patients to play a powerful and active role in the eventual eradication of cancer for future generations.

### Limitations

Decentralized biobanking recognizes the potential for biospecimen research to inform the same individual’s health care. One key limitation relates to the use of “nonhuman subjects research” regulations for biobanking, wherein research proceeds without IRB oversight, explicitly eschewing intervention or interaction with living individuals [42]. The underlying presumption is that there is no duty to return results, as the results are not considered clinically actionable. Our work highlights how this widespread practice results in missed opportunities to fulfill the duty to rescue individuals based on learning from specimens collected during the provision of medical care. In this setting, regulatory considerations (eg, surrounding non-CLIA laboratories) presented early barriers for direct translation of bench research, notwithstanding conceptual appeal and growing sense of moral injury among physicians and scientists. Similarly, anticipation of downstream payor constraints was raised, highlighting potential challenges for justifying coverage of clinical testing or interventions via specimen studies classified as “research-use-only.”

Likewise, while the de-bi app showed the status of specimens regarding whether they were “collected” or “distributed” for research, we were not permitted to reveal specifically “which” scientists were using the specimens, “how” they were used, or “results” thereof. Hence, our technical approach for providing personalized biospecimen feedback focused on providing donors with access to the hub of the biobank database, but did not extend to the research database spokes. Thus, further research and development are needed to determine “how” best to establish linkages between donors and biomedical research, “which” raw data to display for transparency and optimizing access to “results,” while minimizing stress, therapeutic misconception, confusion, and other potential risks.

Importantly, the pilot disproportionately engaged white women with breast cancer in a single region, insufficiently representing the vast array of patients, populations, and conditions that may be impacted by decentralized biobanking. In addition, the piloted app was tailored for biospecimen donors; thus, further research and development must address perspectives of physicians, scientists, and other stakeholders. Future pilots are needed to validate findings with underserved populations and prove feasibility for complex research modalities like patient-derived organoids to advance a robust and sustainable model that can accommodate critical edge cases.

Providing transparency regarding biobank assets via representing personal biospecimens in a biowallet interface empowers patients’ sense of restored ownership. Accordingly, vested interests of biopharma companies, health systems, and academic institutions that profit from no-strings-attached biosample donations represent the foremost barrier to implementing decentralized biobanking. Yet, for our use case, as with most biobanks, 80%-90% of specimens remain in long-term frozen storage, creating financial burdens and undermining progress for the same entities [43,44]. By enabling donors to track their specimens and share benefits from research contributions, a decentralized biobanking platform may address market failure by clarifying ownership and engaging stakeholders with “rails, rules, and tools” engineered to foster collaboration and effective resource usage.

### Futures Directions

The time has come for patients to track their personal contributions to biomedical research: an inevitable consequence of the normalization of mobile computing and internet access for US residents. This approach fits within contemporary calls for transparency to restore trust in health care systems and is thematically consistent with Information Un-Blocking mandates under the 21st Century Cures Act and corresponding international frameworks, such as GDPR [45]. Thus, our first major goal for advancing the decentralized biobanking platform is to empower every patient in the United States to track the samples they donate for research by 2030.

Our proposed person-centered infrastructure will enable a framework for returning benefits to patients, yet will require further innovations and policies to ensure safety and quality. Once biospecimen research may be used to deliver precision medicine solutions, we will be uniquely positioned to harness newfound alignment of stakeholders in an ethically governed biospecimen marketplace. The platform also allows donors to enrich biosample annotation, aid in the linkage of siloed datasets, and improve resource usage via decentralized governance mechanisms. Both nonfungible and fungible tokens may prove useful for promoting efficient collaboration, sharing specimens via decentralized exchange, and providing essential transparency and auditability affordances of blockchain systems. To unlock these benefits, additional research is still required to establish a seamless onboarding experience that minimizes initial friction for users due to the complexities of interacting with blockchain directly. Our work will thoroughly explore emerging standards such as ERC-4337 for account abstraction, which provides programmable flexibility to develop more seamless account creation mechanisms with familiar interfaces. Elsewhere, we discuss ongoing research to overcome other scalability challenges associated with blockchain networks, such as cost optimization strategies to reduce on-chain transaction costs [18].

Including patients as stakeholders is essential to developing ethical marketplace solutions to enhance social value, institutional trustworthiness, and economic sustainability. Further research is needed to define new models for the ethical monetization of biospecimens in partnership with patients and communities. Market validation will require institutional stakeholders to appreciate how they will benefit from democratizing biobank assets. The ability to unlock collaborations between nonprofit biobanks and industry partnerships is a key value proposition—now more than ever in the current research funding environment [46].

Designing our system to optimize resource usage and ensure efficient, effective, and just distribution of benefits is necessary to promote the flourishing of precision medicine as a standard of care [47]. Continuous monitoring and iteration will be required as the model for patient-centered biobanking emerges, is hardened, and developed to accommodate the wide range of potential use cases. Also, there may exist initial hesitancy in the general population about joining new health/research technologies, particularly ones with a seemingly complex financial component. This issue highlights the importance of an initial implementation focused on specimen transparency as an opportunity for education and further research on market designs in tandem with scale-up to optimize acceptability and assuage emergent concerns.

Engaging patients through decentralized biobanking not only enhances transparency and empowerment regarding their biospecimens, but may also foster a greater willingness to share additional behavioral data—such as that obtained from wearable devices. Integrating such real-time, patient-generated health metrics with clinical and biospecimen data could offer clinicians and researchers a more comprehensive view of health trajectories, thereby informing more personalized treatment strategies. Furthermore, as the platform evolves, its capacity to host biobanks and patient cohorts (from multiple institutions) could pave the way for large-scale research studies. In this expanded ecosystem, patients would become full partners, contributing rich, multidimensional data that would otherwise be difficult to amass, ultimately transforming the landscape of precision medicine. Necessary technical enhancements for practical implementation of scalable, decentralized biobanking solutions, such as cost optimization techniques, standardized integration patterns with commonly used laboratory information systems, and research tools, and robust security measures to mitigate risks to users are reported in a study by Sanchez et al [18].

To ensure that decentralized biobanking advances technically as well as ethically, future implementations will incorporate a formal evaluative framework for patient-centered outcomes. While this study emphasized qualitative themes such as dignity, recognition, and empowerment, we have also conducted and are continuing to conduct structured surveys—reported in related publications—that assess patient preferences, perceived agency, trust, and engagement. Prospective implementations will integrate pre-post–survey instruments, as well as behavioral metrics, such as app usage patterns, re-consent rates, and longitudinal participation. These metrics will guide ethical design, inform continuous improvement, and validate the platform’s value from a patient perspective.

Finally, to ensure long-term sustainability, decentralized biobanking must evolve into a multistakeholder platform that delivers value to all participants—patients, researchers, biobanks, and ancillary service providers. This structure enables diversified revenue streams: researchers and institutions may pay for secure, ethically governed access to richly annotated biospecimens and user-enriched metadata; biobanks may subscribe to integrated engagement and tracking services that improve usage and operational efficiency; and third-party service providers (eg, diagnostics, analytics, or consent management tools) may license access or integrate with the platform to expand their offerings. Patients, while not charged, benefit through greater transparency, potential participation in governance, and future models for revenue sharing. Importantly, this structure also aligns with broader health system goals—such as reducing waste, increasing trust, and enhancing data interoperability—and can be designed to meet evolving regulatory frameworks, including GDPR and the 21st Century Cures Act. As such, the decentralized model offers a viable path to sustainability while preserving patient-centricity and ethical integrity.

### Conclusion

Reconnecting patients with their specimens represents a powerful first step for decentralized biobanking that balances ethical obligations, legal precedents, and marketplace demands. Implementing transparency for biospecimen donors regarding their specimens is compatible with current biobank workflows, compliant with deidentification, and necessary for overcoming lethal information asymmetries between bench and bedside. Enabling patients to track their specimens with a decentralized biobanking app represents “low-hanging fruit” with potential for early return on investment by aligning intrinsic incentives and promoting reciprocal research participation, relational integrity, and provenance. Ongoing research seeks to optimize engagement, support seamless return of results, and validate ethical market designs for the decentralized biobanking ecosystem. Reuniting patients and donated specimens may evolve into a holistic digital twin framework, creating a foundation for the future of precision medicine, anchored in building upon existing biobanks while ultimately inviting a sustainable, person-centered Biomediverse.

## Supplementary material

10.2196/73965 Multimedia Appendix 1 Additional tables and figures.

## Abbreviations

BDRR: Breast Disease Research Repository
de-bi: decentralized biobanking
IRB: institutional review board
NFT: nonfungible token

## References

[1] Gross MS, Hood AJ, Rubin JC, Miller RC (2022). Respect, justice and learning are limited when patients are deidentified data subjects. Learn Health Syst.

[2] Cadigan RJ, Juengst E, Davis A, Henderson G (2014). Underutilization of specimens in biobanks: an ethical as well as a practical concern?. Genet Med.

[3] Grizzle WE, Bledsoe MJ, Al Diffalha S, Otali D, Sexton KC (2019). The utilization of biospecimens: impact of the choice of biobanking model. Biopreserv Biobank.

[4] Bledsoe MJ, Sexton KC (2019). Ensuring effective utilization of biospecimens: design, marketing, and other important approaches. Biopreserv Biobank.

[5] Ali M, Naeem F, Kaddoum G, Hossain E (2024). Metaverse communications, networking, security, and applications: research issues, state-of-the-art, and future directions. IEEE Commun Surv Tutorials.

[6] Abhari S, Morita P, Miranda P, Garavand A, Hanjahanja-Phiri T, Chumachenko D (2023). Non-fungible tokens in healthcare: a scoping review. Front Public Health.

[7] Wang Q, Li R, Wang Q, Token C (2021). Non-fungible token (NFT): overview, evaluation, opportunities and challenges. arXiv.

[8] Gross M, Hood AJ, Sanchez WL (2023). Blockchain technology for ethical data practices: decentralized biobanking pilot study. Am J Bioeth.

[9] Gross MS, Miller RC (2019). Ethical implementation of the learning healthcare system with blockchain technology. BHTY.

[10] Meskó B, Kristóf T, Dhunnoo P, Árvai N, Katonai G (2024). Exploring the need for medical futures studies: insights from a scoping review of health care foresight. J Med Internet Res.

[11] Masini E (2006). Rethinking futures studies. Futures.

[12] Stevenson T (2001). The futures of futures studies. Futures.

[13] Sanchez W, Linder L, Miller RC, Hood A, Gross MS (2024). Non-fungible tokens for organoids: decentralized biobanking to empower patients in biospecimen research. Blockchain Healthc Today.

[14] Chen A (2021). The Cold Start Problem: How to Start and Scale Network Effects.

[15] Hood A, Macis M, Mendoza Cervantes D (2024). Privacy, policy, and profits: survey of patient values and preferences for research on de-identified biosamples. Advance.

[16] Pellegrini I, Chabannon C, Mancini J, Viret F, Vey N, Julian-Reynier C (2014). Contributing to research via biobanks: what it means to cancer patients. Health Expect.

[17] Dewan A, Eifler M, Hood A, Sanchez W, Gross M (2025). Building a decentralized biobanking app for research transparency and patient engagement: participatory design study. JMIR Hum Factors.

[18] Sanchez W, Dewan A, Budd E (2025). Decentralized biobanking apps for patient tracking of biospecimen research: real-world usability and feasibility study. JMIR Bioinform Biotechnol.

[19] Gürses S, Troncoso C, Diaz C Engineering privacy by design. IMDEA Software Institute.

[20] Gross M, Miller RC (2021). Protecting privacy and promoting learning: blockchain and privacy preserving technology should inform new ethical guidelines for health data. Health Technol.

[21] van Rest J, Boonstra D, Everts M, van Rijn M, van Paassen R (2014). Designing Privacy-by-Design.

[22] Nouhi M, Heydari M, Goudarzi Z, Shahtaheri RS, Ahmadzaeh A, Olyaeemanesh A (2022). The future effects of COVID-19 on the health system: applying the Futures Wheel method. Med J Islam Repub Iran.

[23] Sirisawat S, Chatjuthamard P, Kiattisin S, Treepongkaruna S (2022). The future of digital donation crowdfunding. PLoS One.

[24] Pant I, Patro L, Sedlander E, Chandrana S, Rimal R (2022). Monitor to innovate with feedback loops: process evaluation protocol for an anemia prevention intervention. Gates Open Res.

[25] Bruun Lorentsen V, Nåden D, Sæteren B (2019). The meaning of dignity when the patients’ bodies are falling apart. Nurs Open.

[26] Gupta V, Eames C, Golding L (2023). Understanding the identity of lived experience researchers and providers: a conceptual framework and systematic narrative review. Res Involv Engagem.

[27] Bengston DN, Westphal LM, Dockry MJ (2020). Back from the Future: The Backcasting Wheel for Mapping a Pathway to a Preferred Future. World Futures Review.

[28] Mamo N, Martin GM, Desira M, Ellul B, Ebejer JP (2020). Dwarna: a blockchain solution for dynamic consent in biobanking. Eur J Hum Genet.

[29] Kuhn T (1962). The Structure of Scientific Revolutions.

[30] Sabharwal K, Hutler B, Eifler M, Gross MS (2025). Decentralized biobanking for transparency, accountability, and engagement in biospecimen donation (in press). J Health Care Law Policy.

[31] Dewan A, Rubin JC, Gross MS (2025). Informed consensus: the future of respect for persons in biomedical research. Am J Bioeth.

[32] Bradfield R, Wright G, Burt G, Cairns G, Van Der Heijden K (2005). The origins and evolution of scenario techniques in long range business planning. Futures.

[33] Slatman J (2012). Phenomenology of bodily integrity in disfiguring breast cancer. Hypatia.

[34] Allen D, Frankel E, Lim W, Siddarth D, Simons J, Weyl EG (2023). Ethics of decentralized social technologies: lessons from web3, the fediverse, and beyond justice, health & democracy impact initiative. Harvard Keneddy School: Ash Center for Democratic Governance and Innovation.

[35] Kuem J, Khansa L, Kim SS (2020). Prominence and engagement: different mechanisms regulating continuance and contribution in online communities. Journal of Management Information Systems.

[36] Nguyen L, Ngwenyama O, Bandyopadhyay A, Nallaperuma K (2024). Realising the potential of digital health communities: a study of the role of social factors in community engagement. Eur J Inf Syst.

[37] Fogg B A behavior model for persuasive design.

[38] Hsiao CH, Chang JJ, Tang KY (2016). Exploring the influential factors in continuance usage of mobile social Apps: Satisfaction, habit, and customer value perspectives. Telemat Inform.

[39] Albalwy F, Brass A, Davies A (2021). A blockchain-based dynamic consent architecture to support clinical genomic data sharing (ConsentChain): proof-of-concept study. JMIR Med Inform.

[40] Weyl G Transforming the present for a fair, decentralized and cooperative tomorrow. https://repositorio.enap.gov.br/bitstream/1/7211/19/Glen%20Weyl.pdf.

[41] Brøgger-Mikkelsen M, Ali Z, Zibert JR, Andersen AD, Thomsen SF (2020). Online patient recruitment in clinical trials: systematic review and meta-analysis. J Med Internet Res.

[42] 46.102 -- definitions for purposes of this policy. eCFR.

[43] Rao A, Vaught J, Tulskie B (2019). Critical financial challenges for biobanking: report of a National Cancer Institute study. Biopreserv Biobank.

[44] Vaught J (2013). Economics: the neglected “omics” of biobanking. Biopreserv Biobank.

[45] Dambrino RJ, Domenico HJ, Graves JA (2023). Unsolicited patient complaints following the 21st Century Cures Act information-blocking rule. JAMA Health Forum.

[46] Seidler D, Winter L, Karlíková M (2024). Bridging financial challenges in young biobanks-funding strategies from the Central Biobank Regensburg. Biopreserv Biobank.

[47] Yang HT, Shah RH, Tegay D, Onel K (2019). Precision oncology: lessons learned and challenges for the future. Cancer Manag Res.

