# Supplementary material for: Decentralized Biobanking Pathway to Precision Medicine: Futures Study
Source: J Med Internet Res. 2025 Dec 1;27:e73965. doi: 10.2196/73965 (PMC12670052; doi:10.2196/73965)
Supplement: Multimedia Appendix 1 [file jmir-v27-e73965-s001.docx]

**Table S1. Details for health data asset taxonomy.**

| - Living models, like organoids and PDX - Tissues, including fresh, frozen or preserved solid biopsies or surgical samples - Fluids, like blood, urine or sweat - Multi-omics like genome, proteome or microbiome - Imaging and wearables, from MRIs or smart-devices like watches, glucose monitors or biometric sensors - Medical records, including doctors notes, labs, pharmacy and claims data |
| --- |

**Table S2. Futures design strategy workshop activities (April 2022).**

| **Day 1**  Set Long Term Goal & List Questions  Ask the Experts  § Learning Health Systems  § Biospecimen Property Law  § Return of Results for Research  § Behavioral Economics/Market Design for Biospecimens  Make a Map  Pick a Target  **Day 2**  Lightning Demos:  Blockchain, Distributed Systems, NFTs  Ethics and Decentralized Biobanking  Organoids and Precision Medicine  § Cybersecurity issues in blockchain/NFTs  § On integrating patient advocates into research activities  § Product development theory and process  § Trust networks, cooperation and social influence in online market design  Organoid Lab and Biobank Tour  Divide or Swarm  Four Step Sketch  **Day 3**  Concept Gallery & Dot Vote (Heatmap)  Speed Critique  Straw Poll & Supervote  Separate Supervote Winners  Rumble or All-in-One  Storyboard / Roadmap |
| --- |

**Table S3. De-bi pilot population demographics.**

**
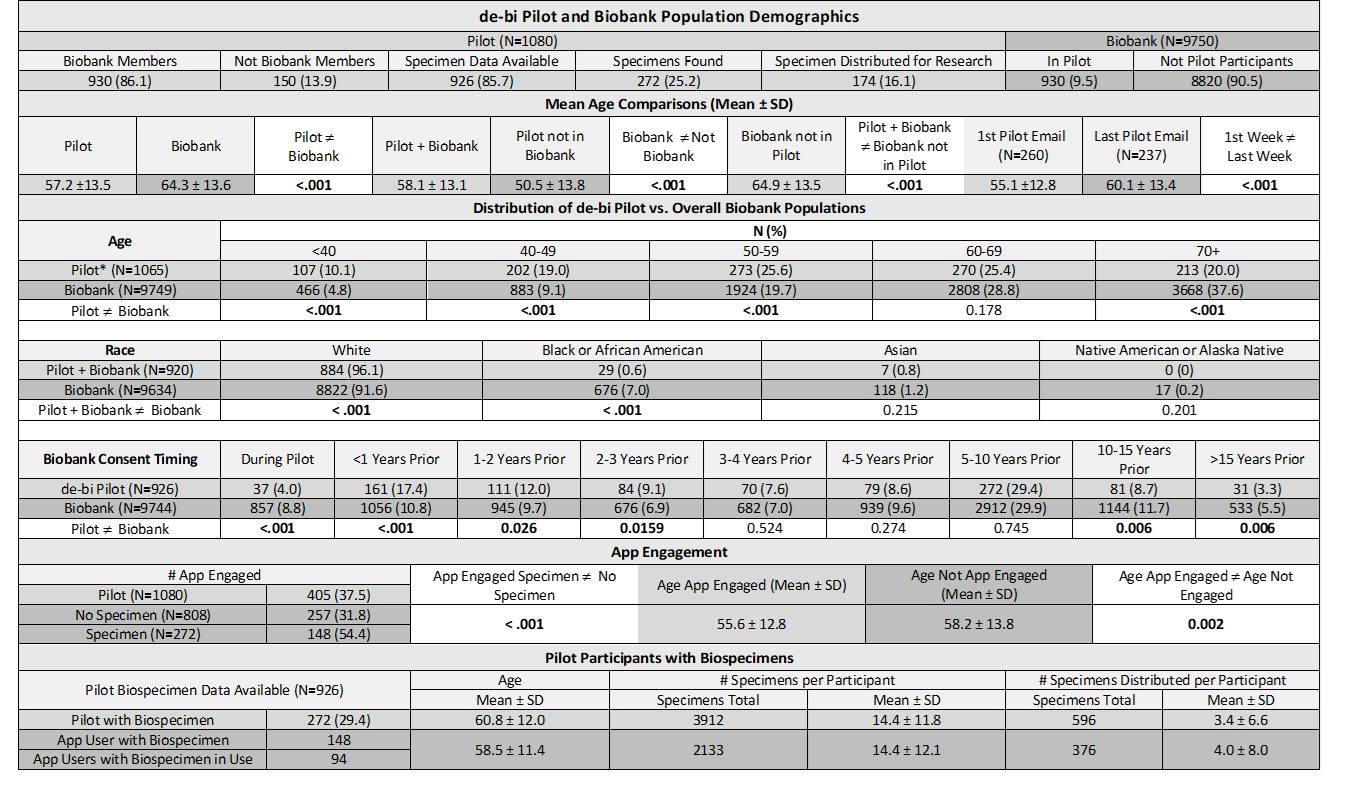
**

¹ (Not equal to)

± (Plus or minus)

SD (Standard Deviation)

< (Less than)

> (Greater than)

*Population demographic proportions were calculated from data with parameters of interest known. Not all entries in Breast Disease Research Repository (BDRR), or biobank, and de-bi Pilot datasets had age data available. Only de-bi pilot participants who were biobank members had race and date of initial biobank consent known. Not all de-bi study participants had the opportunity to test the app. Approximately 200 additional participants received app download invites, but had either not downloaded the app or not created an account before the pilot was completed. Biospecimen data was only available for BDRR members, and some individuals may not have matched to their respective biospecimens due participant typos on the electronic de-bi enrollment consent form and/or institutional database errors that were discovered during data analysis. Of note, approximately one third of BDRR members had contributed specimens to the breast cancer biobank as of the conclusion of the de-bi pilot.

**Table S4. Participatory visioning: direct effects of de-bi pilot.**

| **THEME** | **REPRESENTATIVE QUOTE** | **VISIONING** |
| --- | --- | --- |
| **Dignity** | *The contribution felt disembodied: pun both intended and fitting … the very moment I gave my consent - was that my connection to my own tissue was about to come to an abrupt end in the cold sterility of the surgical suite. Whatever fruit those cells might yield in the lab was destined to be as remote to me as were any youthful illusions of eternal health and invincibility.* | The status quo was depicted as a colorless graveyard, wherein the donor is objectified as a research subject, or “lab rat,” potentiated by the consent process, after which they are locked out and left behind from the subsequent research journey. The sample donation is experienced as a loss, akin to the loss of bodily integrity during the concurrent clinical procedures. |
| **Recognition** | *To me, it’s all about knowledge…I want to be recognized, the acknowledgment of like, yes, that’s my sample.* | Participants envisioned a research system that acknowledges their vital contributions—symbolized as a coin with cellular insignia, that stands up to be counted, aligning with the future vision where donation of samples comes with the dignity of being seen and valued as an individual, not a mere source of scraps. |
| **Understanding** | *Our cells and other samples are sitting somewhere, sort of like Sleeping Beauty, waiting to be kissed. That sounds a little bit passive, because we have to wait for the prince to come in. But in this case, the prince is wearing a white coat…. And instead of a kiss, I get an app. And that app actually allows me to see where my samples are—they're in freezers all over Pittsburgh and the environment around Pittsburgh just waiting for somebody to have an idea to test some new, treatment or approach to identify something about these cells of mine and the other women who are on this journey.* | Understanding was represented as the gateway to the garden of research, wherein participants eyes were opened to the biobanking process, empowered with a digital seat at the table throughout the research journey. By connecting to the ongoing life of their samples through the app, participants unlocked the richness and vibrance of research possibilities their specimens hold, making the scientific process visible and relatable. |
| **Belonging** | *Just knowing that you're not out there alone. You can go onto this app and see that there are other people who have this kind of tumor or diagnoses.* | Within the garden, participants samples were imbued with color and connections to multidimensional communities are unlocked, as individuals with similar diagnoses and samples could find one another, feeling a warmth of common humanity that overcomes stigma and isolation. They envisioned a supportive space, illustrated by flowers and plants growing together, forging a collective, united by shared journeys on the path to discovery. |
| **Ownership** | *Cancer might have compromised my sense of bodily integrity…but the de-bi project…de-bi has radically restored ownership of my tissue to me.* | Participants envisioned a future where they could reclaim a sense of ownership over their bodies and contributions to science. Through the app, the individual remains connected to her samples, wherever they go, envisioned as the “larger than life” patient holding her specimen close to her chest. Restored sovereignty allowed proper inclusion of patients as stakeholders in research, transforming donation from an act of surrender to one of agency, illustrated as increasing density of foliage. |
| **Empowerment** | *With this app, everybody becomes a colleague. … it's my cells, it's me, who's actually pushing the science forward…And it's at those moments when you realize that this is a collaborative effort… So, we as patients are giving as good as we're getting. And those are the magical moments for me.* | Participants envisioned how tracking personal specimens fundamentally changed research from a top-down process to a true collaboration, where patients may be recognized as equal partners in scientific progress. The vision shows patients and scientists walking hand-in-hand down the path to a flourishing garden, where equal stature and direct connection between bench and bedside are highlighted, revealing an ecosystem where impact is amplified by mutual respect and shared purpose. |

**Table S5. Futures Wheel: direct and indirect effects, and implications of de-bi pilot.**

| **DIRECT EFFECTS** | | |
| --- | --- | --- |
| **Empowerment** | *As a participant, I feel I am moving from the role of ‘lab rat’ to research partner.* | By providing a platform for viewing individual and group-level biospecimen activity, donors were included in the research process with a dedicated digital “seat at the table,” setting the stage for further involvement and engagement. |
| **Belonging** | *I can meet other people who have cells like mine… in a two-dimensional space, on an app on my phone...It makes you feel like you're not alone, because we're not.* | The shared biological features of participant tissues and lived experiences of breast cancer treatment and its sequelae binds patients together in a profound, grounding, eternal way that transcends their individual lifetimes. |
| **Recognition** | *With this app, everybody becomes a colleague. … it's my cells, it's me, who's actually pushing the science forward…And it's at those moments when you realize that this is a collaborative effort… So, we as patients are giving as good as we're getting. And those are the magical moments for me.* | Participants highlighted the value of de-bi as a means of sharing knowledge gained from research on donated biospecimens that could inform intergenerational health, enabling continued positive impact from beyond the grave. |
| **Dignity** | *One might say that de-bi has provided for the remembering of my bodily integrity…From radical mastectomy to radical restoration.* | The ability to help other breast cancer patients through the tangible gift of one’s diseased tissues was a significant motivator: the same material object that harmed the individual patient became a tool for healing others. |
| **Ownership** | *“I can’t wait to see what’s being done with my samples!* | Patients were interested in tracking the journey of their personal specimens, sanctified substrates for scientific research with which they had an innate connection and a sense of ownership, reified by visualizing their specimens in the app. |
| **Understanding** | *You can be the victim or be the advocate. With this new app, it’s nice to have your own way of knowing what’s going on with your disease and keep track of your own story. As we look forward to the day when cancer can be managed as a chronic illness and not a death sentence, I’m happy to participate in anything that advances research and patient rights.* | Patients recognized the potential for de-bi to facilitate access to data from studying their own specimens, as well as those with similar disease morphology, which they saw as potentially relevant for informing clinical decisions. |
| **INDIRECT EFFECTS** | | |
| **Community** | *Helping track anyone's samples at this point is going to be crucial to the advancement of medicine… it may not help me right now, but it may help my cancer sisters.* | By creating transparent systems for biospecimen tracking, donors felt a sense of shared purpose and collective benefit, recognizing that their participation could contribute to broader medical advances that support others in the community, even if not directly benefiting themselves. |
| **Legacy** | *I’m writing you on behalf of my late wife… It fills my heart that she is still helping others through this donation… We have two grown children… and it would be a gift to let them know their mom is still helping others from the cross she had to bear.* | Biospecimen donation was seen as a way to create a lasting impact beyond an individual’s life, offering families a sense of meaning and continuity. Through ongoing contributions to research, donors and their loved ones found comfort in knowing their experiences could benefit future generations. |
| **Solace** | *I was especially invested in the opportunity to contribute to breast cancer research. And, given the particularly personal and radical nature of the surgery, it was no small comfort to know that these cells, toxic to me, might prove beneficial to others.* | The opportunity to contribute to research provided emotional comfort and a sense of purpose, transforming a difficult and deeply personal experience into something meaningful. Donors found reassurance in knowing that their challenging journey could potentially help others. |
| **Provenance** | *There was no connection between Ms. Lacks and all the treatments that have ensued. But this way, it's as though I get to step into Henrietta Lacks body, and walk into a lab somewhere, and put my hand on the shoulder of somebody in a white coat, bent over a dish, looking at my cells, trying to figure something out. And so I have my hand on their shoulder, and I can watch, and I can see what's happening.* | By being able to trace the journey of their biospecimens, donors felt a personal connection to the research process, transforming an otherwise invisible contribution into a shared experience. This sense of presence and acknowledgment addressed historical gaps in recognizing the origins and humanity behind research materials. |
| **Appreciation** | *I just embrace every single day that I have. And I'm so, so thankful for research playing such a big role in my life and having a really strong impact.* | Participants expressed deep gratitude for the role research has played in their lives, recognizing how scientific advancements shaped their care and outcomes. This appreciation fueled a desire to give back to research efforts that could help others in similar situations. |
| **Advocacy** | *If patients understand the value of research, then they can be the messengers. They can be the advocates for research… not just research that affects them individually.* | Donors saw themselves as potential advocates, using their experiences to promote the importance of research. By understanding the broader value of scientific work, they felt empowered to encourage others to participate and support research beyond their personal circumstances. |
| **IMPLICATIONS** | | |
| **Network** | *My family is at high risk for breast cancer yet negative for all currently known breast cancer genes. De-Bi would allow my family to pool our tissue samples and offer the history and details of our shared cancer. While samples exist from all the diagnosed breast cancer members of my family (and myself as a previvor), researchers do not have access to the collective pool of our samples, nor do they know they are all from one family with an unknown breast cancer gene.* | By enabling families to link their biospecimens and shared histories, a networked approach could uncover genetic patterns otherwise missed in isolated samples. This collective contribution has the potential to drive discoveries, especially in families with strong but unexplained hereditary risk. |
| **Unity** | *Rarely does science change with one patient. But when you have ten, and ten to the second power, ten to the third power… all coming together in cells that are united in a lab, that's when science changes, and that's when medicine changes, and that's when thriving happens.* | Bringing together biospecimens from many individuals creates a collective force for advancing science and medicine. This sense of unity highlights the power of aggregated contributions to drive meaningful breakthroughs that benefit entire communities, not just individuals. |
| **Gratitude** | *If I'm contributing to any kind of research, if it can help someone else in the future, I’m grateful for that.* | Participants expressed gratitude for the opportunity to contribute to research that could help others in the future. This outlook reflects a broader societal value placed on giving back and advancing knowledge, even without direct personal benefit. |
| **Translation** | *Everybody should be able to have this research done because my tumor is not the same as the person next to me or the person next to them, and to have a standard protocol of treatment doesn't fit in our society.* | Participants emphasized the need for research to translate into personalized care, recognizing that individual differences in disease require tailored treatments. Expanding research access was seen as essential to moving beyond one-size-fits-all approaches and improving outcomes for diverse patients. |
| **Compensation** | *If people are taking our information and using it for their profit, they should share it… [patients] could benefit significantly financially if there was more help and more information.* | Participants raised concerns about fairness and equity, suggesting that if their biospecimens and data contribute to profitable research, donors should share in the benefits. This reflects broader conversations about reciprocity and financial acknowledgment in the research process. |
| **Reciprocity** | *If the researcher asks me for more samples, I'll ask him or her where to go and I will be there.* | Participants expressed a willingness to continue contributing when they felt valued and informed, highlighting the importance of mutual respect and ongoing communication. This sense of reciprocity underscores the need for researchers to engage donors as active partners. |
